# Clinical feasibility of diffusion microstructure imaging (DMI) in acute ischemic stroke

**DOI:** 10.1016/j.nicl.2022.103189

**Published:** 2022-09-09

**Authors:** E. Kellner, M. Reisert, A. Rau, J. Hosp, T. Demerath, C. Weiller, H. Urbach

**Affiliations:** a Department of Radiology, Medical Physics, Medical Center-University of Freiburg Faculty of Medicine, University of Freiburg, Freiburg, Germany; b Department of Stereotactic and Functional Neurosurgery, Medical Center-University of Freiburg, Faculty of Medicine, University of Freiburg, Freiburg, Germany; c Department of Neuroradiology, Medical Center-University of Freiburg, Faculty of Medicine, University of Freiburg, Freiburg, Germany; d Department of Diagnostic and Interventional Radiology, Medical Center-University of Freiburg, Faculty of Medicine, University of Freiburg, Freiburg, Germany; e Department of Neurology and Neuroscience, Medical Center-University of Freiburg, Faculty of Medicine, University of Freiburg, Freiburg, Germany

**Keywords:** Diffusion MRI, Stroke, Diffusion microstructure Imaging, Diffusion Kurtosis Imaging, NODDI

## Abstract

**Background:**

Diffusion microstructure imaging (DMI) is a fast approach to higher-order diffusion-weighted magnetic resonance imaging that allows robust decomposition and characterization of diffusion properties of brain tissue into intra-axonal, extra-axonal, and a free water-compartment. We now report the application of this technique to acute ischemic stroke and demonstrate its potential applicability to the daily clinical routine.

**Methods:**

Thirty-eight patients diagnosed with acute ischemic stroke were scanned using an accelerated multi-shell diffusion-weighted imaging protocol (median delay between onset and MRI scan of 113 min). DMI metrics were calculated and the apparent diffusion coefficient (ADC) derived from conventional diffusion-weighted imaging was used for comparison. The resulting DMI parameter maps were analysed for their potential to improve infarct core delineation, and a receiver-operating characteristic (ROC) analysis was subsequently performed for automated infarct segmentation.

**Results:**

Robust parameter maps for diffusion microstructure properties were obtained in all cases. Within the ischemic tissue, an increase in the volume fraction of the intra-axonal compartment was accompanied by a volume fraction reduction in the other two compartments. Moreover, diffusivity was reduced in all three compartments, with intra-axonal diffusivity showing the highest degree of contrast. The intra-axonal diffusion coefficient maps were subsequently found to perform better than single-shell ADC-derived segmentation in terms of automatic segmentation of the infarct core (area under the curve = 0.98 vs 0.92).

**Conclusions:**

The alterations to the ischemic core detected by DMI are in line with the “beading-model” of non-uniform neurite swelling under ischemic conditions. When compared to conventional single-shell diffusion-weighted imaging, DMI metrics are associated with improved discriminative power for delineating and characterizing ischemic changes. This might allow a more detailed assessment of infarct age, severity of damage, the degree of reversibility, and outcome.

## Highlights

• Feasibility of advanced diffusion microstructure protocol in stroke.
• Acquisition protocol and analysis are fast enough for the acute setting.
• Improved infarct delineation in terms of robustness and sensitivity.
• Interpretation is in line with biophysical models of stroke.

## 1 Introduction

Cerebral imaging in the form of computed tomography (CT) or magnetic resonance imaging (MRI) is critical in acute stroke, given that it forms the basis for further clinical management. In particular, diffusion-weighted imaging (DWI) can detect ischemia-induced changes within minutes of cell death and thus represents one of the most striking and sensitive means of identifying pathologically induced changes (Hjort et al., 2005). Accordingly, a Cochrane review estimated that DWI has a sensitivity of 0.99 (95 % CI, 0.23–1.00), whereas the sensitivity of CT imaging was only found to be 0.39 (95 % CI, 0.16 to 0.69; (Brazzelli et al., 2009)). Although changes in DWI are potentially reversible (Inoue et al., 2014, Labeyrie et al., 2012, Luby et al., 2014, Olivot et al., 2009, Soize et al., 2015), brain tissue with a DWI-derived apparent diffusion coefficient (ADC) of less than approximately 0.620 µm^2^/ms is considered to display the so-called ischemic core. Therefore, even though the use of CT is standard in a clinical setting due to practical and economical reasons, DWI serves both as the ground truth and benchmark for calibration and threshold definition for CT methods (Cereda et al., 2016).

However, the power of diffusion MRI has not yet been fully exploited, as conventional single-shell DWI protocols yield ADC values for a single compartment model only, allowing only a rough approximation of an otherwise complex cerebral microstructure. To overcome this limitation, protocols with multiple b-values based on standard pulsed-field gradients (PFGs) enable the estimation of higher-order terms, such as kurtosis, or the application of multi-compartment models. In fact, diffusional kurtosis has been shown to substantially improve the characterization of tissue microstructure after ischemic stroke in both rodents and humans (Hui et al., 2012, Wang et al., 2019, Weber et al., 2015). Nonetheless, it should be noted that local fiber orientation is an important confounding factor in all modeling approaches that are based on single PFGs. The application of alternative gradient weightings, such as isotropic weightings, double PFG sequences, or q-vector magic angle spinning, allow the underlying microscopic anisotropy to be probed more directly, while also being less dependent on macroscopic fiber organization. Although these alternative techniques seem promising, their practical realisation is hampered by hardware limitations and non-standardized sequences.

To address these limitations, we employed diffusion microstructure imaging (DMI) as a novel multi-compartment analysis technique that uses a Bayesian approach from ordinary single PFG data to disentangle the relative effects on micro-versus macrostructure (Reisert et al., 2017). To date, DMI has been successfully applied to various conditions including idiopathic normal pressure hydrocephalus (Rau et al., 2021), atypical parkinsonism (Rau et al., 2022), peritumoral edema (Würtemberger et al., 2022) and white matter changes in patients in the subacute stage of a SARS-CoV-2 infection (Rau et al., 2022). In general, DMI is based on a variant of the “standard model” of white matter (Jespersen et al., 2007, Novikov et al., 2018, Reisert et al., 2017, Zhang et al., 2012), a three-compartment model comprising an intra-axonal, an extra-axonal and a free-water fraction. Model parameters such as compartmental volume diffusivity are estimated based on signal features that are sensitive to microstructural effects only. In addition, this estimation technique has low measurement requirements and short computation times, and is therefore applicable to an acute stroke scenario. The purpose of this proof-of-concept study is to evaluate the applicability of the DMI approach to an acute stroke setting.

## 2 Methods

### 2.1 Patient cohort

Thirty-eight acute ischemic stroke patients (26 female, mean age 73.3 ± 13.6 years) who received MRI as part of a routine work-up between June 2015 and March 2016 were included in the study (detailed characteristics are presented in Table 1). Patients who were eligible for mechanical thrombectomy according to current guidelines (e.g. no large vessel occlusion (LVO), or the combination of an LVO and a DWI ASPECTS score of 0–4) were excluded from the study to avoid any delay in therapy due to additional sequence acquisition. In 15 patients, i.v. thrombolysis was indicated and initiated during the course of the MRI scan. This study was approved by the local ethics committee (Appl. No. 20–1047), informed written consent was waived.

**Table 1:** Demographics of the considered cohort. (NIHSS - National Institutes of Health Stroke Scale; mRS - modified Rankin Scale).

Patients (n = 38)	Median	IQR	Min	Max
Age (years)	76	18.5	44	94
Sex (female/male)	26 / 12			
Symptom onset − MRI delay (min)	113	30	30	345
NIHSS (max. 32 points)	6	10	0	24
mRS (0–6)	4	1	0	5
Localisation	n = 36 Middle cerebral artery n = 2 Anterior choroidal artery			
Laterality (r/l)	18 / 20			
Lesion size (mL)	4.8	8.3	0.1	148
Thrombolytic treatment (none/ intravenous)	23 / 15			

### 2.2 MRI acquisition

MRI was performed in 24 cases with a 3-Tesla MAGNETOM Tim Trio and in 14 cases with a 3-Tesla MAGNETOM Prisma (Siemens Healthineers, Erlangen, Germany). The diffusion MRI parameters on the Trio-system were as follows: in-plane resolution, 1.5 mm × 1.5 mm; slice thickness, 5 mm; echo time, 102 ms; repetition time, 3400 ms; partial Fourier 6/8. Two b-shells were acquired with b-values of 1000 s/mm^2^ and 2000 s/mm^2^. After every 6th weighting, a b = 0 s/mm^2^ image was inserted. Parameters on the Prisma-system were: in-plane resolution, 1 mm × 1 mm; slice thickness, 5 mm; echo time, 84 ms; repetition time, 3900 ms; partial Fourier 6/8. The diffusion-weighting scheme follows the one recently proposed by the current research group (Reisert et al., 2017) and consists of 28 diffusion weightings hexagonally distributed within a q-ball of radius 2000 s/mm^2^. Total acquisition time was 2:06 min.

### 2.3 Diffusion analysis

Data processing and segmentation were carried out using a local version of the medical imaging platform NORA (https://www.nora-imaging.org). The first pre-processing step comprised denoising (Veraart et al., 2016) and Gibbs-ringing artifact removal (Kellner et al., 2016). DMI metrics were calculated using a recently introduced Bayesian approach (Reisert et al., 2017), which relies on the “standard model” of white matter (Jespersen et al., 2007, Novikov et al., 2018, Reisert et al., 2017, Zhang et al., 2012). The key assumptions behind the standard model are non-exchanging compartments (permeabilities are low) and long diffusion times such that diffusion time dependence can be neglected.

The approach in (Reisert et al., 2017) uses derived features of the signal rather than the raw signal itself. These signal features are invariant with respect to rotation and macroscopic structure and depend exclusively on microstructural tissue properties. In this work, due to the rather fast and low-quality acquisition protocol, we restricted to features of spherical harmonic order 2, which leads to three independent signal features. In comparison, the number of biophysical parameters of the standard model is actually-five. Therefore, we will expect spurious correlations in the DMI parameter maps, because there is simply not enough data to solve the problem exactly. In addition, instead of applying a classical mathematical fit to the maximum a-posteriori estimator (MAP), a supervised machine-learning approach was employed in which the microstructural tissue properties were learned based on a simulated training data set. Thus, the parameters can be calculated for a given measurement in a straightforward manner, given the signal features with a Bayesian estimator. Here, we used a polynomial regressor of order 3 as an estimator. The framework is generally suitable for a large range of microscopic models and dMRI acquisition schemes. The model parameters used to generate the training set for the estimator are universal and were not adapted to the typical parameters of stroke lesions. All parameters were chosen from their possible biophysical range. The only constraint is that D_ax_intra > (D_ax_extra + 2*D_rad_extra), which is necessary due to ambiguities in the standard model for simple single PFG measurements (Novikov et al., 2018, Reisert et al., 2019, Reisert et al., 2017).

**Fig. 1:**
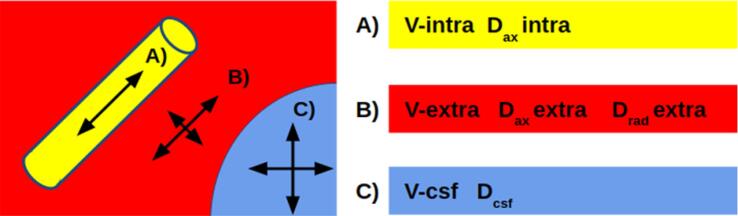
The DMI method is based on a 3-compartment model, where brain tissue is modeled on (A) an intra-axonal compartment, (B) the surrounding extra-axonal compartment and (C) a free-fluid compartment in which water can move freely. The relevant parameters are the volume fractions V of each compartment (adding up to 1), and the relative diffusivity inside the compartments. It is assumed that water can only move along the axonal fibers when it is inside the axons. In the extra-axonal space, there is additional – albeit slight – water movement perpendicular to the fiber orientation, while in the free-fluid compartment, diffusion is isotropic. The diffusion signal is formed by a sum of these compartments, and the parameters are estimated using the Bayesian approach described in Reisert et al. (2017).

The standard model comprises three compartments: an intra-axonal (V-intra), an extra-axonal (V-extra) and a “free-fluid” space (V-CSF), the latter of which accounts for cerebrospinal fluid and perivascular spaces **(**Fig. 1**).** The model is based on the assumption that in the intra-axonal compartment, water can diffuse along axons only, in the extra-axonal compartment it can diffuse both along, and radially to, the orientation of the axons, and in the free-fluid compartment, it can diffuse unrestrictedly in all directions. The diffusivity (D_csf_) of the free-fluid compartment is fixed to 3 μm^2^/ms. This leads to a model with six parameters: The 3 compartmental volume fractions (V-intra, V-extra, V-CSF) and the 3 compartmental diffusivity parameters (D_ax_intra, D_ax_extra and D_rad_extra). The mathematical details of the model are described in (Reisert et al., 2017). The ADC for conventional single-shell DWI was calculated using b = 0 and b = 1000 s/mm^2^ scans yielded by the DMI protocol.

### 2.4 Manual segmentation

We sought to compare the performance of conventional DWI versus DMI for threshold-based infarct core delineation. To control for bias, ground-truth segmentation was manually created based on visual inspection of both conventional DWI and DMI.

For DWI-based segmentation, a threshold of ADC < 0.620 µm^2^/ms (Purushotham et al., 2015) has been used in several studies and served as a guide also here.

**Fig. 2:**
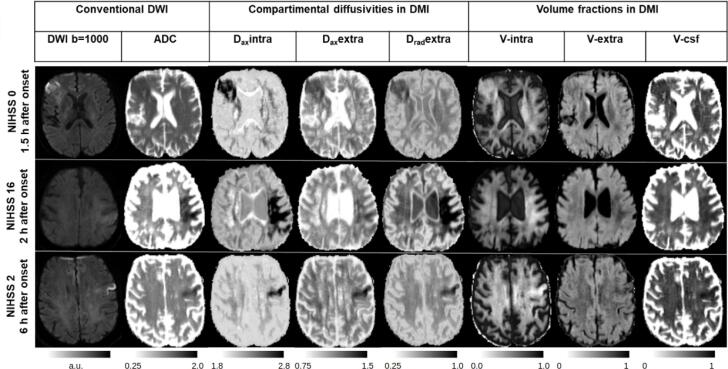
Conventional DWI (left) and DMI maps (right) shown in 3 exemplary cases. Similar qualitative DMI changes were observed within infarct lesions. Diffusivities were decreased in all compartments, with D_ax_intra showing the strongest effects. Regarding the volume fractions, the increase in V-intra is contrary to the decrease in V-extra and V-CSF, indicating net swelling within the intra-axonal compartment. Multi-parametric DMI maps appear to provide a more differentiated, pronounced picture of tissue alterations in stroke compared to that elicited by conventional DWI.

For DMI segmentation, the major focus was on D_ax_intra mapping, which showed the strongest level of contrast in the infarct core upon visual inspection and is most closely related to conventional ADC (see Fig. 2). To exclude false positives, special care was taken to identify motion artifacts and speckle noise during manual segmentation. Putative diffusion abnormalities in the basal ganglia, usually caused by a low signal, were intentionally disregarded. To assure that all lesion voxels were included, interpolation of the selected voxels was performed whenever appropriate, to ensure that at least several voxels per slice were segmented.

### 2.5 Statistical analysis

Segmentation performance in the infarct core was evaluated using receiver-operating characteristics (ROC), which compares sensitivity and specificity for a range of different thresholds. Voxels from all subjects were pooled together to generate the ROC curve. Due to the lack of ground truth available for the infarct cores, comparison was performed in a pair-wise fashion with four different settings used as ground truth: A. manual DWI-based segmentation, B. DMI-based segmentation, C. the intersection of the DWI- and DMI-based infarct core or D. the union of the DWI- and DMI-based infarct core, where settings C. and D. act as two additional ‘consensus’ ground truths. Prior to thresholding, the images were brain-stripped using a mask generated with the tool FSL-BET (Jenkinson et al., 2012) to exclude noise from non-brain-voxels. ROC curves were generated, and the area under the curve (AUC), the maximum of Youden’s statistics (J = sensitivity + specificity − 1), and the optimal threshold where J is maximized were calculated.

Additionally, we report descriptive statistics of all parameters of the DMI model within the lesion and a corresponding area on the contralateral side. Therefore, we used the intersection (C) lesion mask and used SPM12 to mirror (co-registration with a x-flipped version of the b0-image) the lesion to the contralateral side. We also used a brain mask to clip all CSF-voxels from the flipped lesion mask.

## 3 Results

**Fig. 3:**
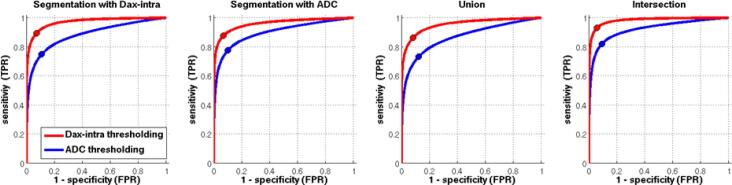
ROC analysis for infarct core segmentation based on DMI and ADC. Manual expert segmentation based on both DWI-derived ADC and DMI-derived D_ax_intra, their union or their intersection were used as the ground truth and compared to full threshold-based infarct core segmentation with various thresholds. The resulting curves indicate that DMI-derived D_ax_intra has a higher discriminative power for infarct core segmentation. The dots correspond to thresholds derived from the Youdens’ index.

**Fig. 4:**
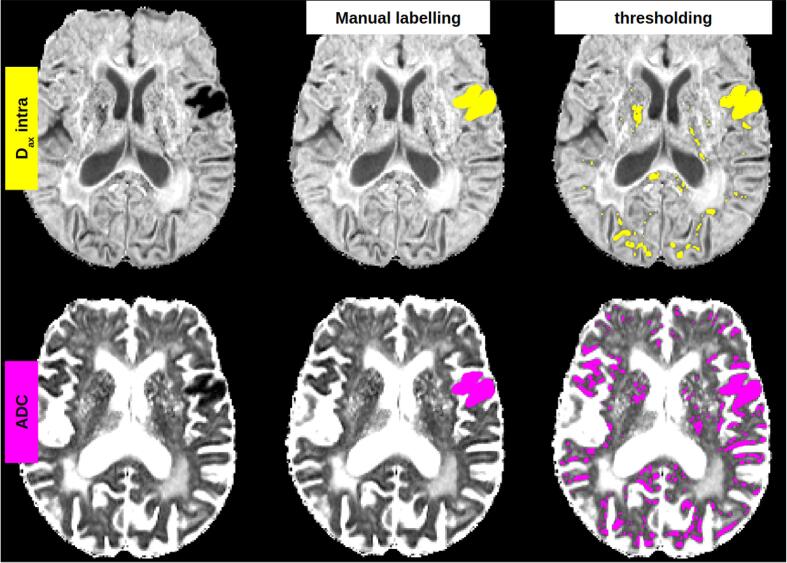
Comparison of DMI segmentation with ADC-based segmentation of the infarct core in a 77 year-old female patient 145 min after symptom onset. The DMI-derived intra-axonal diffusivity (D_ax_intra) generally has a sharper, more discriminative contrast than the ADC map (note the more distinct gray/white matter junction). ROC analysis was performed by comparing the ground-truth segmentations for both contrast techniques (middle column) as well as their union and intersection (not shown) with various thresholds (example in right column). D_ax_intra-based thresholding is clearly more discriminative and has less noise (i.e. false positives) than ADC-based thresholding. Note the increased diffusivities in several white matter regions due to the severe leukencephalopathy of the patient.

**Fig. 5:**
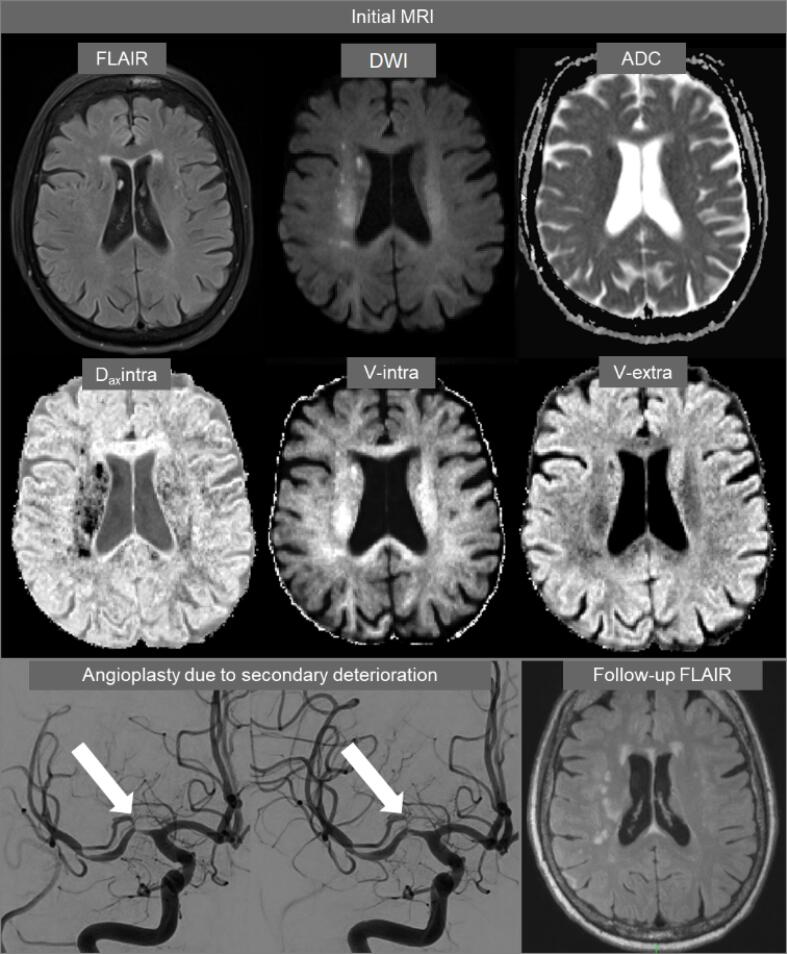
A 78-year-old woman presented with fluctuating paresis of the left arm and dysarthria due to high-grade stenosis of the right middle cerebral artery since 11 h. FLAIR showed no signal alterations, ADC revealed subtle ischemic changes in the deep border zone between perforating MCA branches and deep medullary arteries. A DWI (averaged over b ≥ 1800) shows already clear signal alterations, and DMI reveals a clear reduction in intra-axonal diffusivity (D_ax_intra), an increase in V-intra and concurrent reduction in the volume of V-extra. Following clinical deterioration after the MRI scan, the patient was treated with angioplasty. Note that the extent of ischemic changes on FLAIR follow-up is more consistent with the axial intraaxonal diffusivity map than ADC is.

Both conventional DWI and DMI parameters were successfully calculated in all 38 patients. Fig. 2 shows representative examples of the DMI-derived quantitative parameter maps alongside a conventional single-shell, DWI-derived mean image and apparent-diffusion-coefficient (ADC). Application of the DMI method revealed an increase in the volume fraction of V-intra, in contrast to a volume fraction reduction in the remaining 2 compartments (V-extra, V-CSF). Reduced diffusivities were observed within all compartments. Upon visual evaluation, the D_ax_intra map showed the strongest degree of contrast between the infarct core and the preserved tissue. Therefore, we compared D_ax_intra-based automated segmentation of the infarct core to a conventional ADC-based approach. Here, ROC analysis revealed that DMI had a better performance level compared to that of conventional DWI, (Fig. 3 and Table 2) whereby the maximum sensitivity and specificity at an optimal threshold of D_ax_intra (0.93/0.94, AUC = 0.98) was higher than in conventional ADC (0.82/0.90, AUC = 0.92). The optimal threshold for D_ax_intra hits 2 µm^2^/ms almost perfectly. For ADC the threshold is slightly below 0.7 µm^2^/ms. Fig. 4 shows a representative example of the superiority of automatic D_ax_intra-based segmentation of the infarct core. Fig. 5 depicts the case of a 77-year-old woman who presented with fluctuating left-sided arm paresis and dysarthria due to high-grade, right-sided middle cerebral artery stenosis. She was treated with angioplasty after she clinically deteriorated 1 h after the MRI scan. Acute D_ax_intra predicted the extent and configuration of the delimited FLAIR-lesion yielded by a follow-up MRI much better than that of acute DWI or ADC maps.

**Table 2:** ROC measures for DMI and conventional ADC (**AUC**: area under the curve; **Jmax**: maximum of Youdens’ index; **Threshold**: optimal threshold at max of Youdens’ index; S**ens/Spec**: sensitivity/specificity). For all ground-truth variants, DMI-based D_ax_intra showed the best performance (also see ROC curves in Fig. 3). Units for D_ax_intra and ADC thresholds are µm^2^/ms.

Ground truth	D_ax_intra				ADC			
	AUC	Jmax	Threshold	Sens/Spec	AUC	Jmax	Threshold	Sens/Spec
Segmentation with D_ax_intra	0.97	0.82	2.02	0.89/0.93	0.88	0.64	0.689	0.75/0.89
Segmentation with ADC	0.96	0.81	2.00	0.88/0.93	0.90	0.67	0.683	0.78/0.90
Union	0.95	0.78	2.02	0.86/0.92	0.87	0.61	0.697	0.73/0.88
Intersection	0.98	0.87	1.99	0.93/0.94	0.92	0.73	0.678	0.82/0.90

**Fig. 6:**
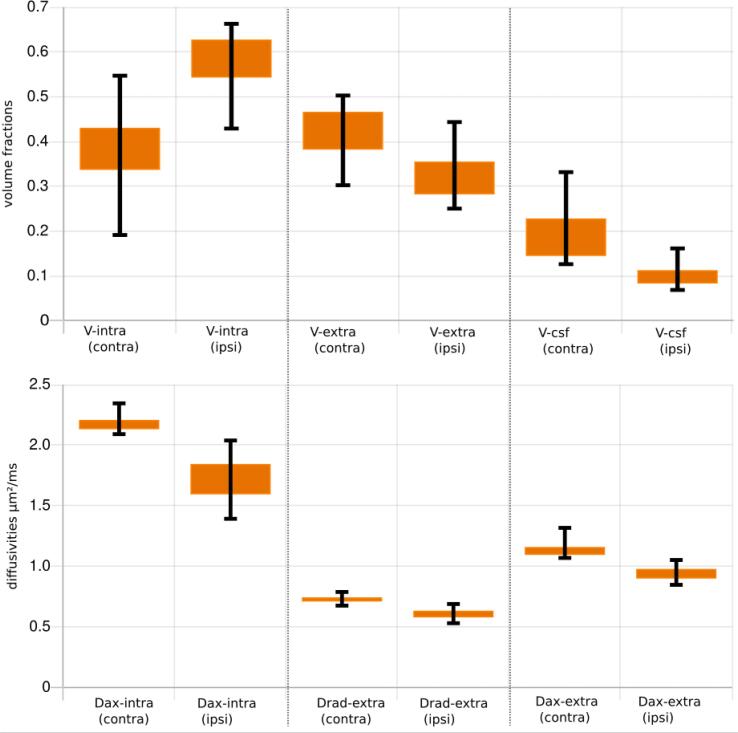
Statistics over the whole group of subjects of all model parameters within the lesion mask. For comparison of mean values of the mirrored lesion mask (contra) on the healthy hemisphere is also shown. Errorbars indicate the lower 5%-percentile and the upper 95%-percentile.

Fig. 6 depicts descriptive statistics of all DMI parameters of the ipsi- and contralateral lesion mask.

## 4 Discussion

We report the successful application of DMI in 38 patients with acute ischemic stroke. Based on our results, DMI (especially D_ax_intra) provides a contrast between ischemic and preserved tissue that is superior to that of conventional DWI, leading to the higher accuracy of automatic threshold-based segmentation of the estimated infarct core. This approach may further allow more accurate predictions of final stroke volume and configuration. Due to our ultra-fast protocol (2:06 min), this technique is highly applicable to the daily clinical routine, even with respect to the time-critical nature of acute stroke imaging.

The potential of advanced dMRI techniques to characterize ischemic tissue has already been explored in other studies. In line with our findings, increases in both the sensitivity and specificity of higher-order diffusion metrics have been demonstrated in both animals and humans using diffusion kurtosis imaging (DiBella et al., 2022, Hui et al., 2012, Liu et al., 2019, Spampinato et al., 2017, Umesh Rudrapatna et al., 2014). In contrast to DKI, the Bayesian approach in DMI allows for a highly robust estimation of parameters, even with just a few diffusion directions. Most importantly, however, DMI can directly yield not only the physical parameters of the model, but also the volume fractions and diffusivities of each compartment. Moreover, our results are in line with a study that used NODDI metrics to estimate microstructure properties in ischemic tissue (Wang et al., 2019) which showed significantly larger absolute percentage changes than those derived from DTI and DKI.

However, the NODDI approach was originally developed for healthy tissue and requires stabilization by strict *a priori* constraints, hence making it less applicable to pathologically altered brain tissue.

There are several limitations associated with the interpretation of our data. The cross-sectional character of our dataset (except for the single patient described in Fig. 5**)** particularly needs to be taken into account, since the FLAIR-delimited ischemic infarct could not be used as ground truth and secondary growth of the final infarct core could not be ruled out. To overcome this problem, we performed crosswise validation using both conventional ADC and DMI. The baseline ADC threshold used in this study is just ‘one’ of the thresholds appearing in literature. We used 0.620 µm^2^/ms, because it is one of the most common choices (Albers et al., 2016; Purushotham et al., 2015), and it is used by several automatic processing tools. However, the optimal ADC threshold for the present study (∼0.680 µm^2^/ms) was somewhat higher. The lack of a follow-up investigation for the definition of an appropriate ground truth likely explains this deviation. Finally, another limitation of our study is that the considered cohort is not fully representative of an acute ischemic stroke population, because patients which underwent thrombectomy were excluded.

Interestingly, a volume shift to the intra-axonal compartment accompanied by reduced diffusivity was observed within the infarct core (see Fig. 2). This pattern fits well with the concept of a non-uniform, focal enlargement of axons due to osmotic imbalance, which has been defined as axonal beading based on mathematical models and sciatic nerve preparations (Budde and Frank, 2010). In this setting, axons are thought to undergo a form of net swelling (increased V-intra) that has a non-uniform, bead-like configuration, which changes the overall axon morphology to periodically, reoccurring barriers along the length of the axon. These barriers lead to a reduction in intra-axonal diffusivity. In the context of conventional single-shell DWI, these changes are summarised into the ADC map as a single averaged parameter. Although the model of axonal beading primarily covers the white matter, the intra-axonal compartment has been also adopted for dendritic neurites within the gray matter (Genç et al., 2018). Whether DMI parameters are differentially changed within ischemic white vs gray matter needs to be investigated in future studies. As a further remark in this context, the dMRI standard model used by DMI has been developed for white matter, as it implies no exchange between the one-dimensional compartment (V-intra) and the extra-axonal (V-extra) and CSF space. While this assumption is well justified by the myelin-sheaths in white matter, it might not be valid in gray matter. In fact, recent studies have been pointing at a non-negligible exchange between dendrites and extracellular space, on the scale of 20–60 ms (Jelescu et al., 2022), or even <10 ms (Olesen et al., 2021). Here, we can not sharply distinguish between V and intra (neurites) and V-extra (cells and extracellular matrix). A final remark can be made on the signal features, where we restricted the model to information up to second order spherical harmonics, which was, due to the quality of the data, necessary.

## CRediT authorship contribution statement

**E. Kellner:** Conceptualization, Data curation, Formal analysis. **M. Reisert:** Conceptualization, Data curation, Formal analysis. **A. Rau:** Conceptualization, Data curation, Formal analysis. **J. Hosp:** Writing – review & editing. **T. Demerath:** Writing – review & editing. **C. Weiller:** Supervision, Writing – review & editing, Project administration. **H. Urbach:** Supervision, Writing – review & editing, Project administration.

## Declaration of Competing Interest

The authors declare that they have no known competing financial interests or personal relationships that could have appeared to influence the work reported in this paper.

## Data Availability

The authors do not have permission to share data.
